# A comparative study of the established methods and evaluation of rat trauma models

**DOI:** 10.1002/ame2.12479

**Published:** 2024-10-22

**Authors:** Zhenmin Sun, Jia Sun, Gang Su, Ruohan Wang, Zhaohui Zhai, Feng Yu, Yuli Li

**Affiliations:** ^1^ Qingdao Hospital University of Health and Rehabilitation Sciences, Qingdao Municipal Hospital Qingdao China; ^2^ Shandong Second Medical University Weifang China; ^3^ State Key Laboratory of Marine Resource Utilization in South China Sea, Hainan Provincial Key Laboratory of Research on Utilization of Si‐Zr‐Ti Resources, College of Materials Science and Engineering Hainan University Haikou China

**Keywords:** age of the rats, healing speed, modeling, rat trauma, skin tension unloader

## Abstract

**Background:**

Scientific animal models are indispensable for studying trauma repair. This work aimed at establishing a more scientific rat trauma model by studying different rat trauma models caused by different trauma numbers, locations, and trauma attachment tension unloaders and rat age.

**Methods:**

A four‐trauma self‐upper, lower, left and right control model; a two‐trauma self‐trauma bare and ring control model; and a young and old rat trauma model were created to evaluate the condition of these traumas.

**Results:**

In the four‐trauma self‐control model, the healing status of the upper proximal cephalic trauma was better than that of the lower proximal caudal trauma, whereas there was no significant difference between the left and right trauma. The healing rate and postwound condition of the trauma with a ring control in the two‐trauma model were better than those of the bare side. The healing speed of the old rats was slower, and the amount of extracellular matrix in the subcutaneous tissue after healing was significantly lower than that of the young rats.

**Conclusion:**

The double trauma with a ring is a more scientific and reasonable experimental model. There is a significant difference between young and old rats in the wound healing process. Therefore, the appropriate age of the rats should be selected according to the main age range of the patients with similar conditions in the clinical setting being mimicked.

## Graphical abstract

This study established a more scientific model of back trauma in rats. We discuss the limitations of the commonly used rat trauma model, propose a double‐trauma model with rings on the back of rats, and discuss the advantages of this model. The age selection of rats was also discussed.

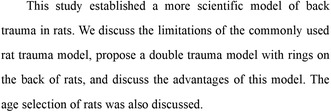

## 1 INTRODUCTION

Currently, the commonly used animal models for skin trauma are mice, rats, rabbits, and pigs, of which rats are the most widely used. A scientific and reasonable animal model can reduce the interference of other factors besides experimental factors and can better explore the correlation between trauma healing and experimental methods. This work used rats as experimental subjects. Three series of experiments were carried out to investigate the appropriate location and number of trauma fabrications, the effect of trauma peripheral attachment rings on trauma healing, and the age selection and representativeness of model rats. Based on a large amount of related references and research results, this paper discusses the limitations of the traditional modeling methods and puts forward a more scientific trauma modeling method by considering aspects like trauma location, trauma number, trauma attachment ring treatment, and rat age selection.

Currently, most researchers use total cortical excision to mimic skin trauma in animal experiments,^1^, ^2^, ^3^, ^4^ but the location and number of trauma vary. For example, some scholars use six trauma^5^ or six‐like trauma^6^ in small animal models, some studies use four trauma,^7^, ^8^, ^9^ some studies use two trauma,^10^, ^11^, ^12^, ^13^ and some researchers use a single trauma.^14^, ^15^, ^16^, ^17^, ^18^, ^19^, ^20^ Therefore, the location and number of wounds, especially for self‐control experiments, must be determined immediately. The varying blood supply at different wound sites may have a great effect on the wound healing rate. Therefore, it needs to be studied whether the wound model used will have an impact on the scientific evaluation of the experimental results.

Some papers have reported that investigators have used full cortical excision followed by direct application of ointment or dressing, regardless of skin tension, which has the advantage of better simulating the natural conditions of human wounds during healing.^8^, ^17^, ^21^, ^22^ Some researchers have used metal or silicone fixation on the outer edge of the wound to reduce skin tension and prevent stretching of the wound. Tension unloaders are now commonly used to accelerate wound healing and reduce scar formation.^23^, ^24^, ^25^, ^26^, ^27^

Finally, the selection of rats based on age and physiological status varies from one researcher to another, with some differentiating by body weight and using male rats of 200–250 g^1^, ^5^, ^7^, ^22^, ^26^, ^28^, ^29^ and others differentiating by rat age and using rats aged 6–8 weeks.^28^, ^30^, ^31^ However, many chronic difficult‐to‐heal wounds in the clinic are in the elderly, such as decubitus ulcers and diabetic foot, which are highly prevalent in the elderly. Therefore, we believe that rats aged 6–8 weeks cannot accurately simulate the skin healing state of the elderly, and the selection of rat age is also one of the focal points of this article.

## 2 MATERIALS AND METHODS

### 2.1 Animals

**FIGURE 1:**
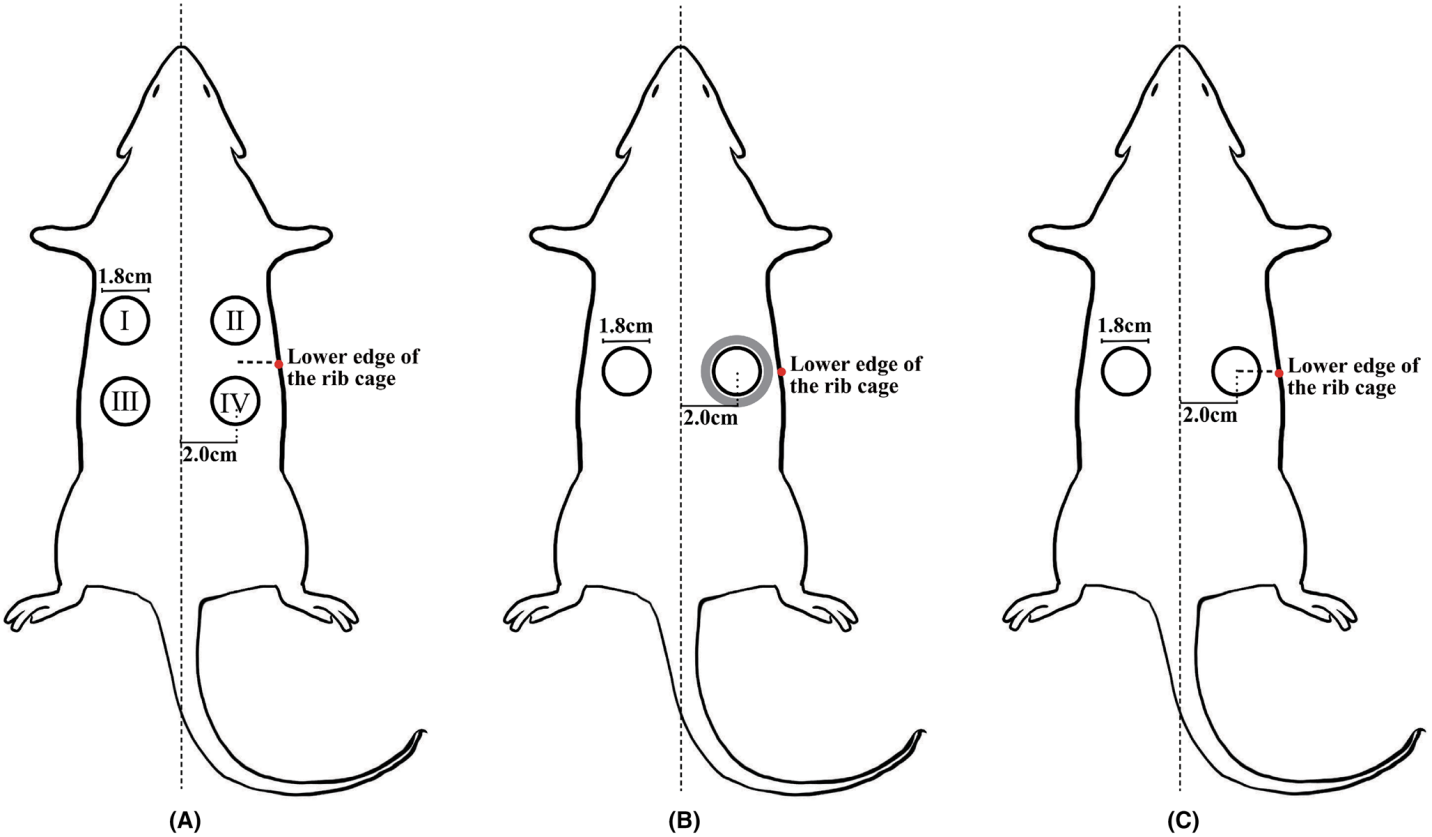
The models used in three experiments. (A) Experiment 1. (B) Experiment 2. (C) Experiment 3.

Healthy male Sprague–Dawley (SD) rats (age: 6 and 48 weeks, mean body mass at 6 weeks: 360 ± 10 g, and mean body mass at 48 weeks: 400 ± 20 g) were purchased from Beijing Viton Lever Laboratory Animal Technology Co. The 6‐week‐old rats were used for the four‐trauma self‐control model in experiment 1, as shown in Figure 1A; the 6‐week‐old rats were also used for the self‐controlled double‐wound model in experiment 2 (Figure 1B). The double‐trauma model for the young rats (6 weeks old) and the old rats (48 weeks old) was carried out in experiment 3, as shown in Figure 1C. Rats of the same age were grouped using the Excel table random number grouping method. Five rats were used in each experiment, and the wound healing areas were recorded on days 3, 7, 10, and 14. The rats were executed on day 14, and the wound tissues were fixed and stained with hematoxylin–eosin (H&E) to evaluate tissue healing and pathological changes in the wounds.

### 2.2 Model preparation and treatment

The rats were anesthetized; the hair on their back was removed. In the four‐trauma control model (experiment 1), four 18‐mm‐diameter circular skin lesions were formed along the contour of the circle. In the two‐trauma model (experiment 2), two round full skin defect traumas (18 mm in diameter) on each side of the rat's dorsal surface opposite to the inferior border of the rib cage were formed. One of them was naked, and the other was controlled using a 22‐mm‐diameter circular silicone ring attached to the periphery. In experiment 3, the two‐trauma model was utilized to evaluate the healing speed difference between the 6‐week‐old young rat group and the 48‐week‐old old rat group using the same trauma size and location as described in experiment 2.

### 2.3 Result collection

The general conditions of the rats, including rat weight, wound area, and wound healing status, were observed and recorded at each dressing and wound excision. All the rats were killed on day 14, and the traumatic skin tissue was excised and preserved.

### 2.4 Statistical analysis

All wound images were transferred to a computer and changed to a tagged information file format extension using Adobe Photoshop Elements 40. The wound area was measured using ImageJ, a special size analysis software. The change in wound size was expressed as a percentage of the original wound size. SPSS 19.0 statistical software was used for data analysis. The results were expressed as mean ± standard deviation (*x* ± *s*). If they conformed to normal distribution and *χ* ^2^, independent samples *t*‐test or analysis of variance (ANOVA) was used. If they did not conform to normal distribution or *χ* ^2^, nonparametric test was performed, and the differences were considered statistically significant at *p* < 0.05.

## 3 RESULTS

### 3.1 Analysis of the number of experimental animals

A total of 20 SD rats were used in the experiment, and the wound healing rate was recorded at different time points. The wound tissues were stained with H&E on day 14. The rats did not die in the middle of the experiment, and there were no large differences in weight change in the same group. All rats were included in result analysis.

### 3.2 Documentation of wound healing process

**FIGURE 2:**
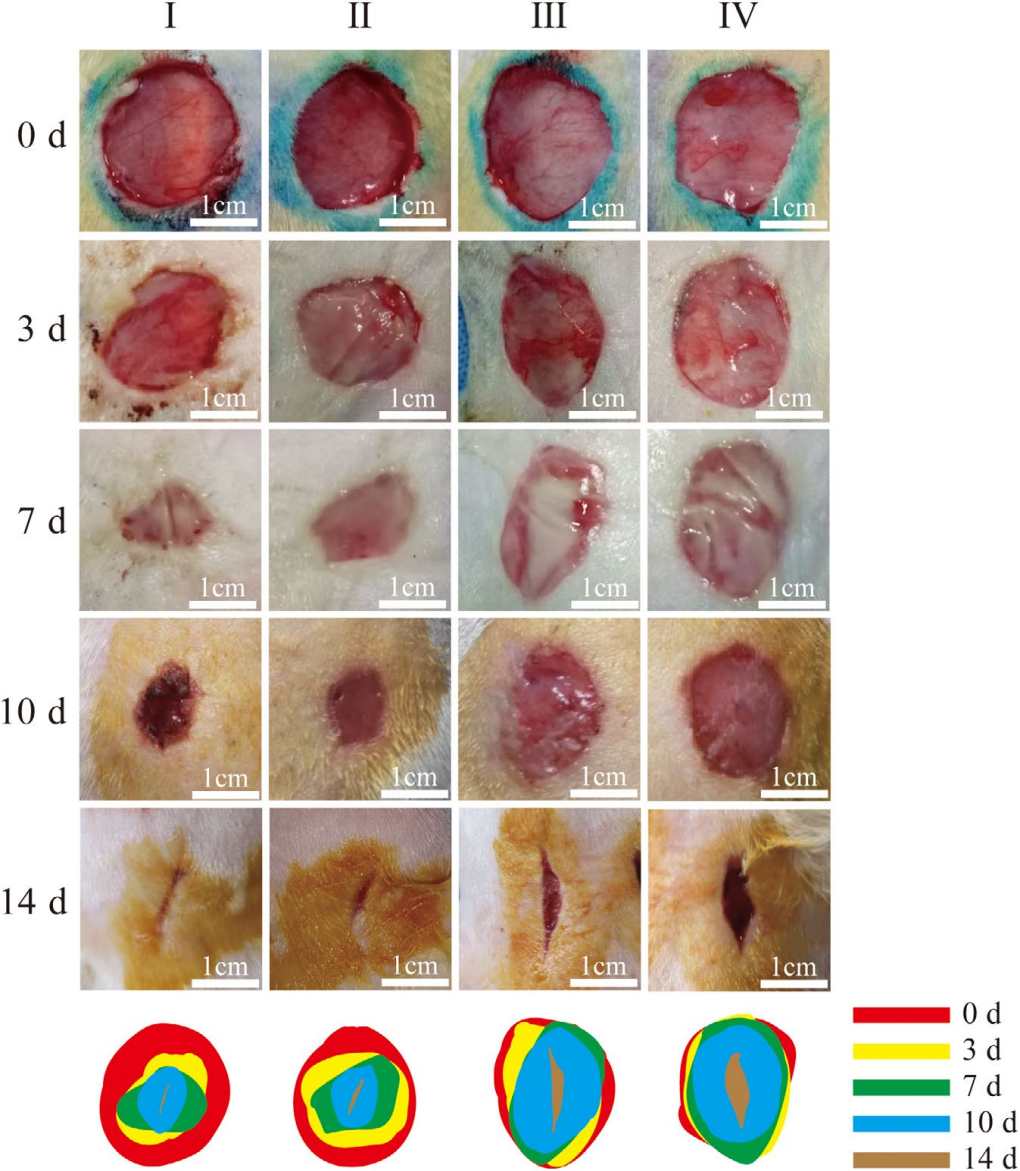
Representative images of four‐trauma groups of rats in the wound healing process in different locations and at different time points.

**FIGURE 3:**
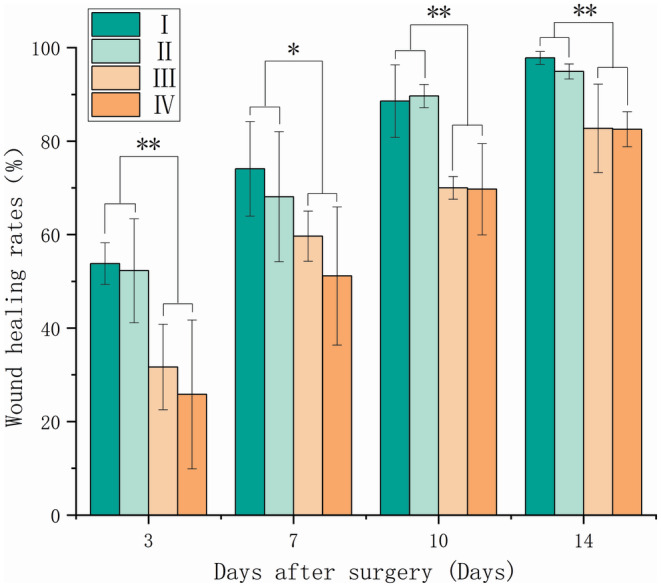
Healing rates of different locations of wounds in the four‐trauma model, *p < 0.05, **p < 0.01.

**FIGURE 4:**
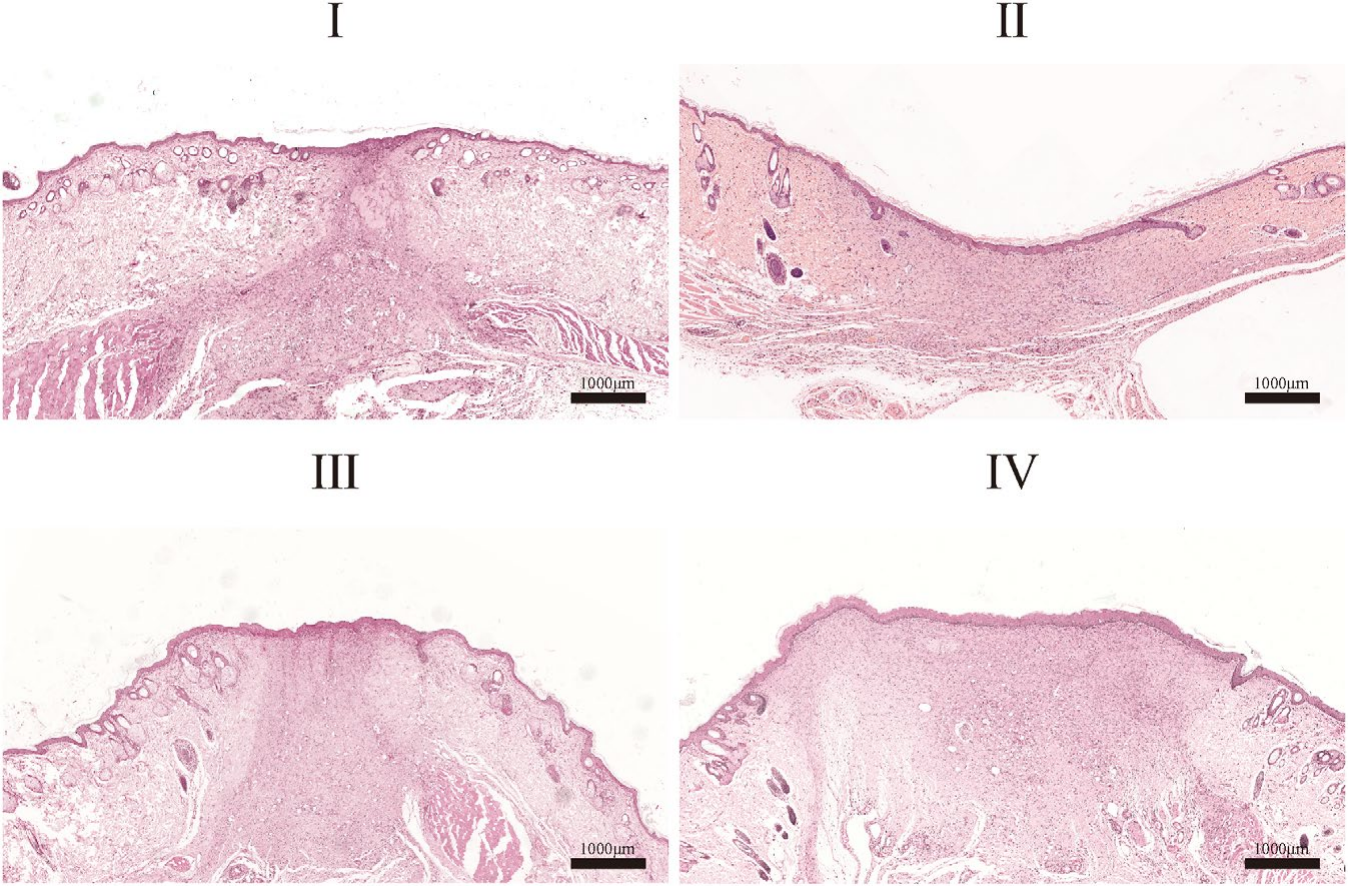
Representative images of H&E (hematoxylin–eosin) staining after 14 days at different locations in the four‐trauma model.

Figure 2 shows the wound healing process at different locations in the four‐trauma group at different healing time points. Figure 3 shows the statistics of the healing rate of the four wounds, and Figure 4 shows the representative images of H&E staining after 14 days for the four wounds. Figures 2 and 3 show that the healing status of traumas I and II on the proximal cephalic side and that of traumas III and IV on the proximal caudal side are similar. However, the healing status of traumas I and II on the proximal cephalic side was better than that of traumas III and IV on the proximal caudal side. Figure 4 shows that re‐epithelialization was nearly complete in all experimental subjects. There was more tissue proximal to the cephalic side in close proximity to normal tissue, indicating a greater healing capacity proximal to the cephalic end than proximal to the caudal side. In addition, all the four traumas healed, resulting in the shape of a willow leaf or a linear shape under exposed conditions, which was consistent with the results of experiment 2 on the exposed side.

**FIGURE 5:**
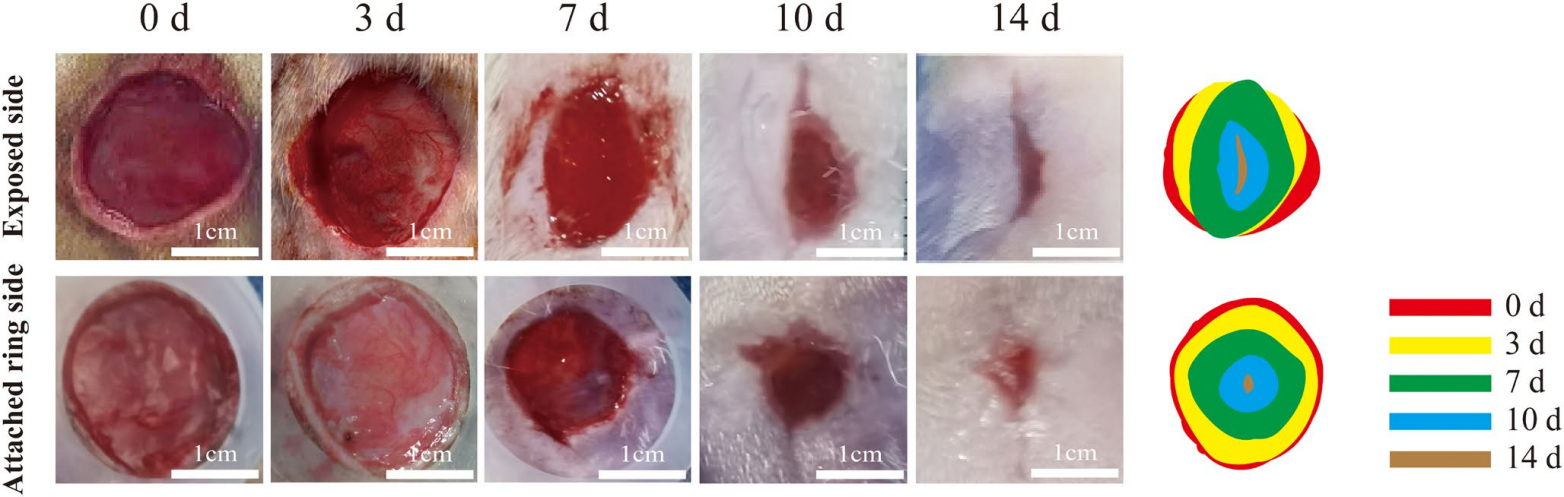
Representative images of the healing process of the wound on the bare side and the attached ring side.

**FIGURE 6:**
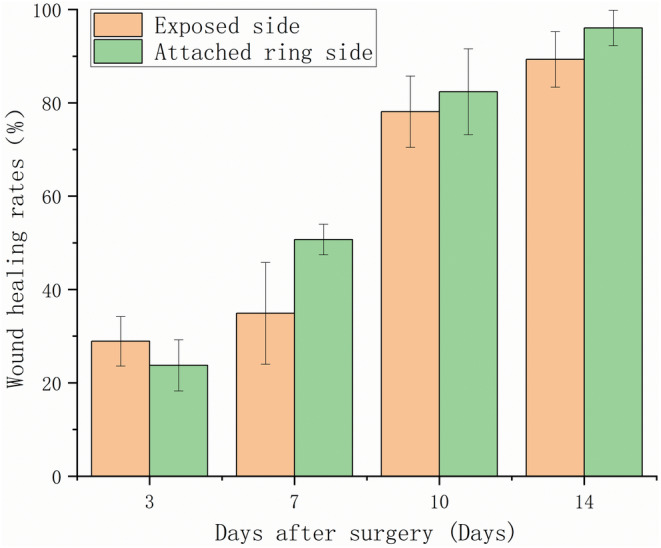
Wound healing rate of the bare side and the attached ring side.

Figure 5 shows images of the bare and the attached ring groups at different healing time points. The shape of the exposed side of the wound was oval, extending to both sides of the head and tail, and gradually changed to the shape of a willow leaf during the healing process under the action of skin tension, eventually forming a long strip‐shaped scar. The wounds on the side with an attached ring gradually healed in a circular shape and eventually formed small scars, which are less obvious than those on the bare side and more visually appealing to the naked eye. The statistical results of the wound healing rate in the bare and the attached ring groups are shown in Figure 6. Combining with Figure 5, we can conclude that the wound healing rate on the attached ring side was slower than that on the bare side during the first 3 days, but then the rate increased from days 3 to 7 and continued to increase.

**FIGURE 7:**
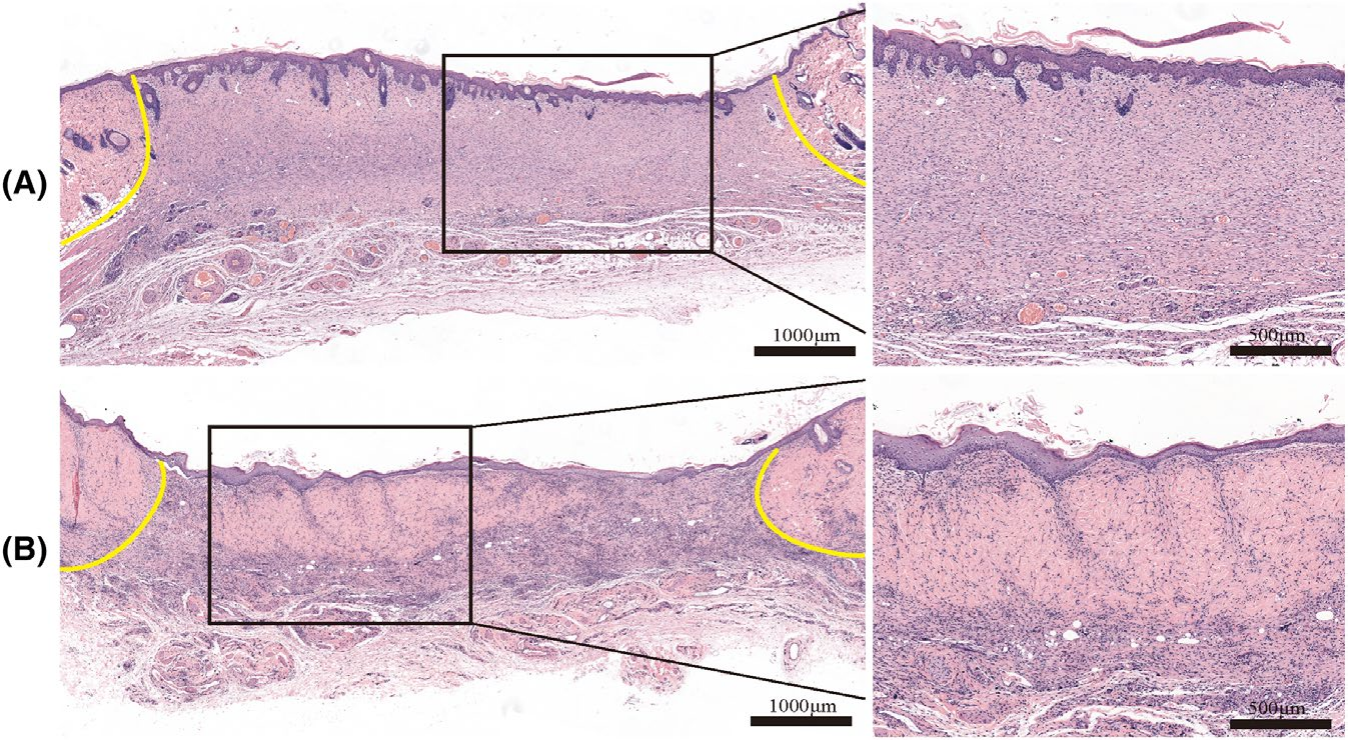
Representative images of H&E (hematoxylin–eosin) staining of the bare side and the attached ring side of the wound at 14 days. The boundary between normal tissue and healing tissue is indicated by the yellow line. (A) Bare side. (B) Attached ring side.

The H&E staining results shown in Figure 7 show that wound re‐epithelialization was completed in all experimental individuals with adhesive rings attached to the edges, and covered the entire wound area. The wound had entered the tissue remodeling phase, with some of the new tissue structures in the dermis approaching healthy tissue. Although the re‐epithelialization process was also completed in the bare group, the histomorphological structure of the dermis was still in the early stage of repair and differed greatly from that of normal tissue. This indicates that the healing rate of the attached ring group was significantly faster than that of the bare group. In addition, the migrating and regenerated epithelium of the attached ring group was smoother and more uniform than that of the bare group, and visually the area of the scar of the ring group was smaller than that of the bare group, which was flatter and visually pleasant.

**FIGURE 8:**
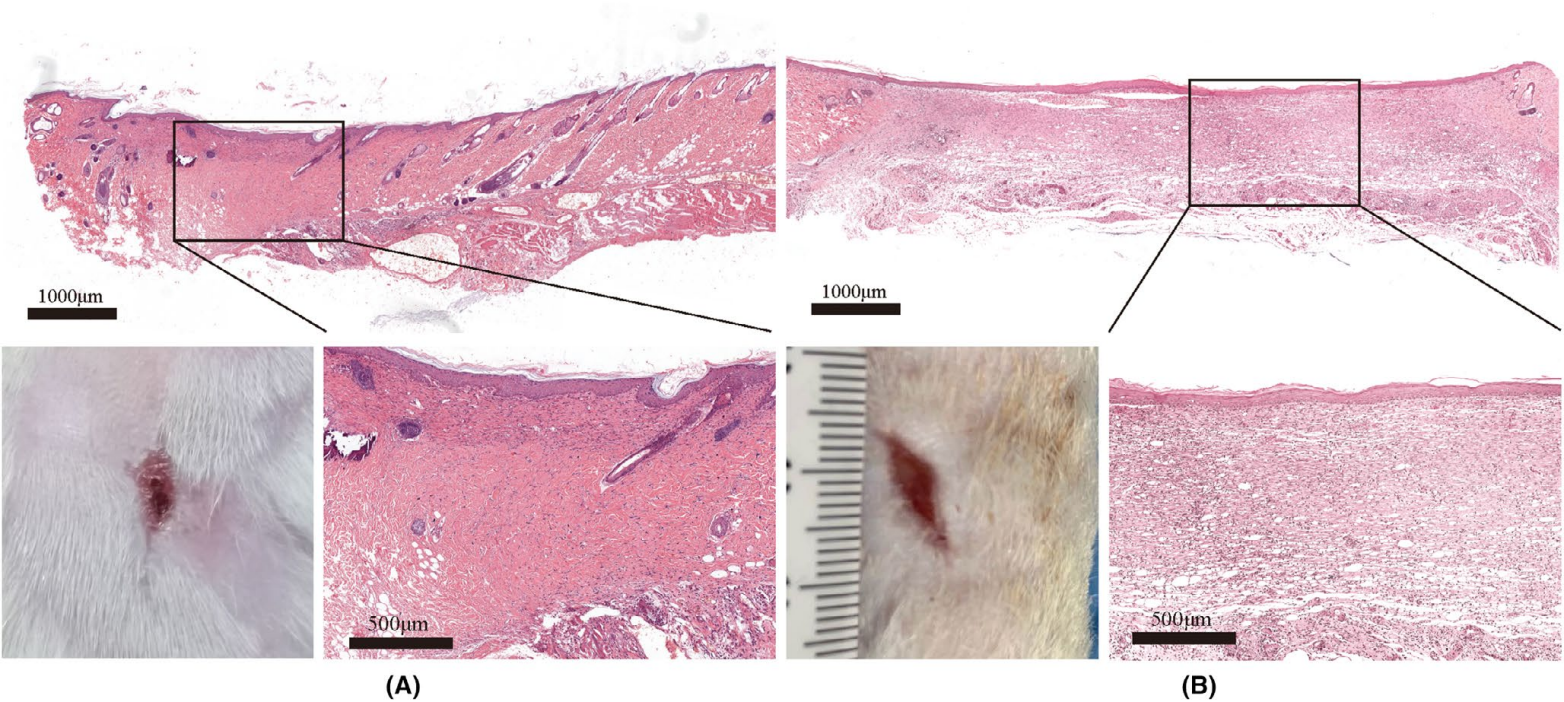
H&E (hematoxylin–eosin) staining of 14‐day trauma and representative images of traumas in (A) the young group and (B) the old group.

**FIGURE 9:**
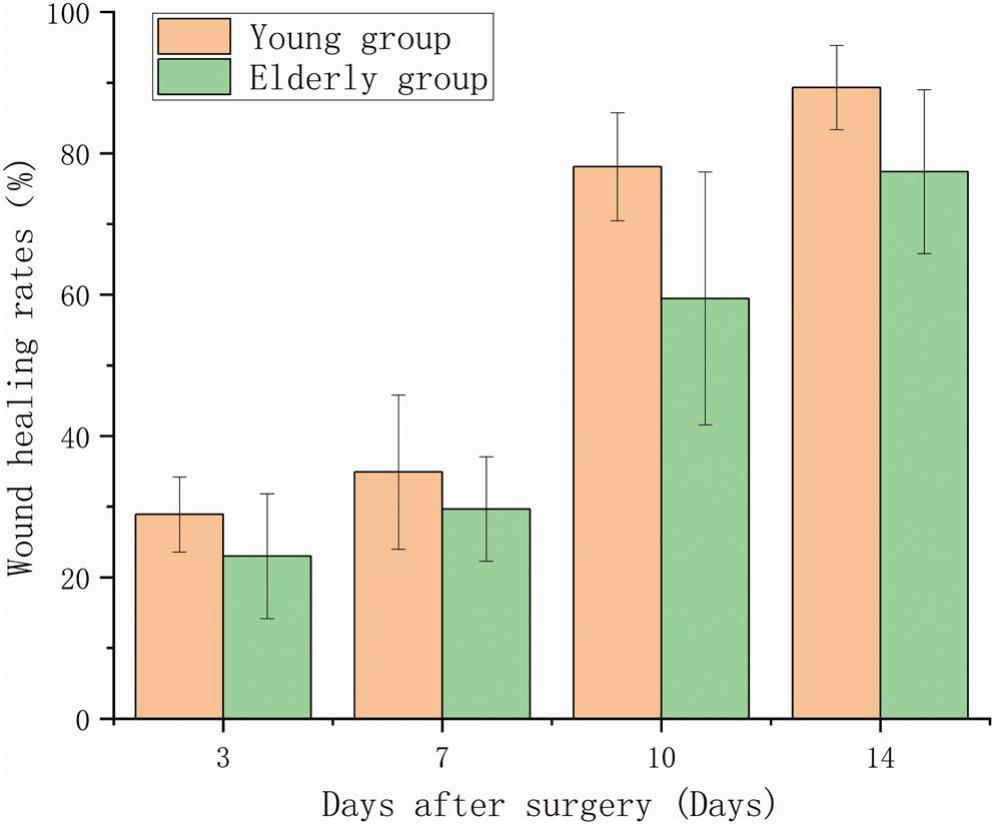
Comparison of the wound healing rate between the young and the old groups.

**FIGURE 10:**
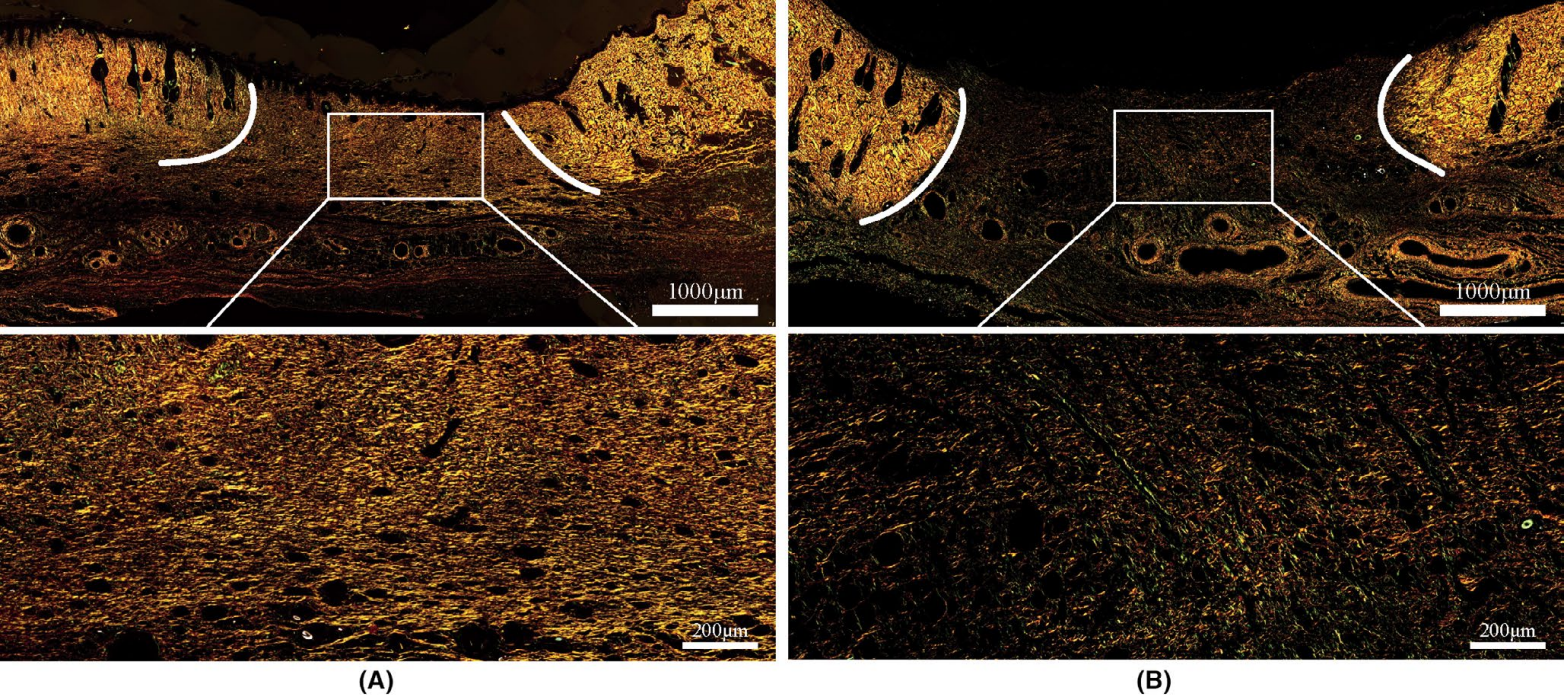
Sirius red–stained images of wounds after 14 days of healing in (A) the young and (B) old groups. The boundary between normal tissue and healing tissue is indicated by the white line.

Figures 8 and 9 show that the wound healing rate of the young group was faster compared with that of the old group, and H&E staining shows that the young group had already entered the tissue remodeling stage, whereas the old group had only completed re‐epithelialization, and the dermis was still in the state of tissue crawling. The Sirius red staining results in Figure 10 show that on day 14, the collagen in the wound area of the young group closely resembled normal tissue. The collagen was densely arranged, primarily consisting of type I collagen and less type III collagen. Additionally, the degree of wound healing was high. The collagen fibers in the old group were sparsely arranged. Type I collagen fibers and type III collagen fibers were mixed, and the proportion of type III collagen was relatively high, indicating that it is still in the primary stage of healing.

## 4 DISCUSSION

The design of animal experiments should be based on the needs of the experiment and supported by scientific theory. The physical and genetic properties of the SD rats are similar to those of humans, which increases the predictive accuracy of trauma models based on SD rats when applied to human trauma. SD rats also have a strong adaptability and disease resistance, growing rapidly to reach an appropriate weight and age in a short period. Additionally, the care and management of SD rats is easy; they rarely develop spontaneous tumors and have a relatively stable genetics, all of which contribute to the consistency of experimental data. For more challenging diabetic wounds, SD rats are highly sensitive to establishing a diabetes model, and the SD rat diabetes model exhibits good stability and repeatability, making it an excellent experimental model. The numerous advantages of the SD rat make it one of the most frequently used breeds in current animal research. In the selection of rat age, we designated 6‐week‐old male SD rats as the young group. During the entire experimental process, the age of the rats was between 6 and 8 weeks, which is the early adulthood period. At this time, the rats have a moderate body size, making them easier to handle, and their hormonal and immune systems are relatively stable, reacting to trauma similar to humans. In the choice of sex, male rats have more stable hormone levels than female rats, which helps maintain consistency in the rats during the experiment. The choice of trauma size needs to be based on experimental needs, physiological characteristics of the experimental animals, and ethical considerations. For experiments that require observation of the recovery process of the same batch of rats, the trauma should be relatively small to minimize the pain of the rats and ensure their survival rate. In this experiment, an 18‐mm wound was used, which was basically healed by the end of the experiment on day 14, and the whole healing process could be observed completely.

In experiment 1, the difference in wound healing between the proximal cephalic side and the proximal caudal side may be attributed to variations in blood supply. The formation of neovascularization is essential for effective wound healing, because it is beneficial to deliver nutrients and maintain a stable oxygen supply, providing sufficient blood flow to allow cell proliferation and tissue regeneration.^32^ Based on the statistical results, we can conclude that there are differences in the wound healing status at different positions along the vertical axis between the head and tail sides of the rat. However, there is no significant difference between the left and right sides at the same position along the vertical axis. The four‐trauma model can reduce the error caused by individual differences and reduce the use of experimental animals. However, the different healing efficiencies of the upper and lower wounds caused by blood supply and other factors necessitate further investigation of the experimental significance. Therefore, we think that when establishing the rat's own control model, a double‐trauma model should be established on the rat's back with the spine as the symmetry axis. If the four‐wound model or other multi‐wound models are used, only left–right controls should be utilized, and controls with different vertical positions should not be employed. In addition, due to the existence of blood supply and other influencing factors, the healing rate at the edge of the wound is different in the same wound. Therefore, when slicing tissue, it is not appropriate to simply use the unhealed part at the center of the wound. It is more reasonable to record the initial position of the wound for the final accurate cutting. This can prevent the wrong judgment of the original trauma location and epidermal crawl when analyzing the experimental results. It is obvious that the specimen will fail to cover the original trauma location, resulting in incomplete acquisition of experimental data if the excision range is too small.

Skin tension can not only cause scar formation but also is closely related to the pattern and degree of scar hyperplasia.^33^ Reducing periwound skin tension is a key strategy in preventing scar formation.^34^ Unlike human skin, the skin of rodents, including rats, is elastic but lacks adhesion to underlying structures, and skin closure is achieved by traction through myofilament or femoral muscle stimulation.^35^, ^36^ Skin tension has a great effect on wound healing in rats, which makes unloading of periwound skin tension a necessary condition for experiments. Experiment 2 used an adhesion ring as a tension unloader to reduce the effect of skin tension. The experimental results showed that the healing rate of the attached ring side was slower than that of the bare side in the early stage of wound healing. Because the wound with an attached ring is forced to be pulled into a circle initially, that is the shape with the largest area of the same perimeter. It is also worth noting that the inner diameter of the adhesive ring or other types of pressure unloaders should be at a distance from the edge of the wound. The reason is that the wound will enlarge immediately after it is made due to the tension of the skin and its elasticity. The small inner diameter of the ring may cause the adhesive contaminating the wound and affect the healing. In our experimental group, the wound healing rate was significantly delayed when a 20‐mm inner‐diameter ring was applied to an 18‐mm wound and was significantly improved when a 22‐mm ring was applied to an 18‐mm wound. The wound healing rate on the attached ring side was higher than that on the bare side after 3 days. Because the attached ring side of the wound effectively reduces the interference of skin tension, the epidermal and dermal crawl and stretch in all directions at the edge of the healing wound were more uniform and balanced. The H&E staining results in Figure 7 showed that the trauma repair had entered the tissue remodeling phase. It means that the granulation tissue was gradually replaced by normal connective tissue, and the degree of recovery of the trauma was high. Although the wound on the bare side heals faster in the early stage, the total healing time is longer than that for the side with the ring and the wound is often stretched during the healing process and more likely to form scars. Meanwhile, in addition to the skin tension and skin wrinkles, the rats often bend due to pain, which will also cause the wound to be stretched and deformed along the direction of tension, resulting in a longer healing period.^37^ The healing skin shows undesirable manifestations such as folds, unevenness, and scars. And the experimental results show that the adhesion ring used in this experiment can indeed play the role of a skin tension unloader and effectively improve the healing of rat skin. This is more in line with the current clinical treatment, because tension‐relieving bands are now often used in hospitals to treat wounds. We aim to better simulate the actual clinical treatment process. Therefore, when establishing the rat trauma model, the fixation ring should be attached to the outer edge of the trauma.

Collagen produced by fibroblasts is critical for granulation tissue formation. Mast cells and macrophages are key inflammatory cells in the process of wound healing. There were fewer fibroblasts, mast cells, and macrophages in the dermis of aged skin compared with young skin. A decrease in the number of these cells leads to delayed wound healing.^38^ With age, the level of collagen and Extracellular matrix (ECM) components such as Glycosaminoglycans (GAGs) decreases,^39^ and the cell cycle of fibroblasts in the tissue is significantly prolonged, both of which result in delayed healing. Skin collagen is mainly composed of type I and type III collagen. Type I collagen plays a supporting role in skin structure, whereas type III collagen increases in the early stage of skin wound healing and plays an important role in collagen reconstruction.^40^ Sirius red staining results in Figure 10 show that there is a large gap in the level of collagen production and the ability of wound repair between old and young mice. Therefore, the aged mice should not be treated as young mice in the study of wound healing. Some studies have suggested that the overexpression of Matrix Metalloproteinases (MMPs), mitochondrial dysfunction, and the depletion of stem cell banks may be the reasons for the impaired skin healing ability in aging.^41^, ^42^ It also leads to thinning of the epidermis after healing. This phenomenon was also observed in the old rats in this experiment. In the comparative experiments of rat age, we found that some researchers in the study of the simulated human diabetic rat model used male rats <20 weeks old that had not yet entered the mating period. But the good clinical age of human diabetes is ≥45 years, which is at least equivalent to more than 48 weeks in rats.^43^, ^44^, ^45^, ^46^ Therefore, these simulated clinical animal models are inaccurate; 6‐week‐old young rats had a significantly faster wound healing rate than 48‐week‐old rats. The healing ability of the skin in aged rats is degraded due to age. In this case, the modeling approach for aged rats remains to be investigated. Therefore, when modeling age‐related diseases, the age of the rats should be determined based on the type of disease and the age of predilection. The physiological differences caused by age should be carefully considered to ensure the relevance of the experimental model to humans.

This experiment demonstrates that the limitations of the widely used trauma models, such as the four‐ and six‐trauma models, depend on the different wound healing abilities at different positions, and the consistency of the experiment needs to be studied. Another widely used single‐trauma model does not prevent the influence of individual differences on the consistency of experimental results. Compared with other wound models, the double‐trauma model with rings on the back of rats can effectively reduce the wound deformation caused by the difference between the structure of the rat skin and human skin, and can better simulate the healing process of human wounds. However, the current model can compare only two sets of experimental conditions at a particular time and is not convenient for comparing multiple sets. The empirical tips on the many details mentioned in this paper are also capable of enhancing the scientific and ethical validity of experimental results. The study of diabetic wounds has become a significant area of research. This experiment employed SD rats as the majority of current diabetic animal models to establish a healthy rat trauma model, which is conducive to further exploring the establishment of a scientific diabetic trauma model. The study of the differences in wound healing between different rat ages also suggests that the selection of the age of experimental animals is important in the study of age‐related diseases such as diabetes. The study of the relationship between rodent and human ages is quite limited, especially for SD rats. A significant number of researchers fail to consider the effect of rat age in their studies on age‐related diseases. The results of the experiments indicate that further research on the relationship between rat age and human age is necessary for the study of age‐related diseases. The improvement in the skin tension unloader is also one of the necessary research directions in the future. This includes the exploration of strategies to more accurately simulate human wounds and minimize wound damage. Additionally, the development of novel biomaterials that act as drug carriers and facilitate wound healing while maintaining the integrity of the wound is a crucial area of investigation.

## 5 CONCLUSIONS

When studying trauma repair, it is more scientific and reasonable to use a double‐trauma model with rings on the back of rats for comparison. Meanwhile the choice of rat age should be based on different disease models.

## AUTHOR CONTRIBUTIONS

Yuli Li supervised the project and worked on experimental design, provided financial support and administrative support, and was responsible for the final approval of the manuscript. Zhenmin Sun and Jia Sun performed the majority of the experiments and wrote the paper. Feng Yu assisted in writing the manuscript and manuscript revisions. Zhaohui Zhai provided technical support for the experiments. Gang Su and Ruohan Wang contributed to the animal experiments. All authors have made substantial contributions to the article and approved its submission.

## FUNDING INFORMATION

This work was supported by funds from the Key Program of Shandong Provincial Natural Science Foundation (ZR2020KE018), the National Natural Science Foundation of China (52003068), the Shandong Provincial Natural Science Foundation (ZR2022MH211). We would like to thank all the reviewers who participated in the review.

## CONFLICT OF INTEREST STATEMENT

The authors declare that they have no conflict of interest.

## ETHICS STATEMENT

This study was performed in strict accordance with the recommendations in the Guide for the Care and Use of Laboratory Animals published by the Shandong Second Medical University. The protocol was approved by the Committee on the Ethics of Animal Experiments of Shandong Second Medical University (Permit Number: 2021SDL053). All surgery was performed under isoflurane gas anesthesia, and every effort was made to minimize suffering.
